# In silico pipeline for GSK 3β inhibitor discovery in Alzheimer’s disease using pharmacophore screening, docking, ADME filtering, and MD validation

**DOI:** 10.1038/s41598-026-56505-6

**Published:** 2026-07-23

**Authors:** Mahmoud S. Elkotamy, Mohamed K. Elgohary, Ahmed S. Alkotami, Mohamed M. Eldesouki, Zainab M. Elsayed, Amr A. Mattar, Mahmoud F. Abo-Ashour, Haytham O. Tawfik, Wagdy M. Eldehna, Hatem A. Abdel-Aziz

**Affiliations:** 1 Department of Pharmaceutical Chemistry, Faculty of Pharmacy, Alsalam University, Tanta, 31511 Egypt; 2 Pharmaceutical Chemistry Department, Faculty of Pharmacy, Egyptian Russian University, Badr City, Cairo 11829 Egypt; 3 Neurology Department, Faculty of Medicine, Tanta University, Tanta, Egypt; 4 Scientific Research and Innovation Support Unit, Faculty of Pharmacy, Kafrelsheikh University, Kafrelsheikh, 33516 Egypt; 5 Department of Pharmaceutical Chemistry, Faculty of Pharmacy, El Saleheya El Gadida University, El Saleheya El Gadida, Egypt; 6 Department of Pharmaceutical Chemistry, Faculty of Pharmacy, Tanta University, Tanta, 31527 Egypt; 7 Department of Pharmaceutical Chemistry, Faculty of Pharmacy, Kafrelsheikh University, P.O. Box 33516, Kafrelsheikh, Egypt; 8 Department of Applied Organic Chemistry, National Research Center, Dokki, Cairo 12622 Egypt

**Keywords:** GSK-3β, Alzheimer’s disease, Virtual screening, Pharmacophore, Molecular dynamics, Computational biology and bioinformatics, Drug discovery

## Abstract

Glycogen synthase kinase-3β (GSK-3β) is a key therapeutic target for Alzheimer’s disease, but identifying safe, brain-penetrant inhibitors remains difficult. This study aimed to discover novel CNS-active GSK-3β inhibitors using a rigorous multi-tier computational pipeline. The workflow combined ligand-based and structure-based pharmacophore modeling, virtual screening of the ZINCPharmer database, AutoDock Vina docking, ADME and blood-brain barrier (BBB) filtering with SwissADME, toxicity prediction using ProTox-3.0, and validation by 100-ns molecular dynamics simulations with MM/GBSA and MM/PBSA free energy calculations. Pharmacophore screening with a ≤ 1.0 Å RMSD cutoff identified 1,085 ligand-based and 36 structure-based hits. After docking and developability filtering, two BBB-permeant candidates were prioritized: **SB1**, a structure-based hit (predicted LD_50_ = 2500 mg/kg, toxicity class 5), and **LB1**, a ligand-based hit (predicted LD_50_ = 521 mg/kg, toxicity class 4). Molecular dynamics confirmed stable binding for both compounds. MM/GBSA analysis showed favorable binding free energies for **SB1** (-27.68 kcal/mol) and **LB1** (-25.74 kcal/mol), both surpassing the co-crystallized reference (-8.75 kcal/mol). These findings identify **SB1** and **LB1** as promising, safe, and brain-penetrant GSK-3β lead compounds for experimental validation in Alzheimer’s disease.

## Introduction

Alzheimer’s disease (AD) represents a progressive neurodegenerative disorder and is the predominant cause of dementia among older adults. Clinical symptoms initiate with mild memory lapses and executive dysfunction, ultimately advancing to significant cognitive and functional impairment^1^. Individuals generally survive approximately 4 to 8 years post-diagnosis, with some cases extending up to 20 years, indicative of the disease’s slow and progressive nature. In 2021, approximately 57 million individuals worldwide were diagnosed with dementia, with 60–70% of these cases attributed to Alzheimer’s disease^2^. Alzheimer’s disease is a prominent contributor to mortality, currently ranking as the seventh leading cause of death globally, thereby imposing a significant societal burden^3^. In 2022, approximately 6.5 million Americans aged 65 and older were diagnosed with Alzheimer’s disease dementia, a figure anticipated to double by 2060 without significant advancements^4^.

Glycogen synthase kinase-3β (GSK-3β) is a widely expressed serine/threonine kinase that has significant implications in the pathogenesis of Alzheimer’s disease. In the Alzheimer’s disease brain, GSK-3β expression and activity are significantly elevated^5^. GSK-3β phosphorylates numerous substrates pertinent to neuronal function, and various studies associate its dysregulation with the characteristics of Alzheimer’s disease. GSK-3β is a key regulator of tau hyperphosphorylation and the formation of neurofibrillary tangles (NFTs). Tau possesses approximately 40 phosphorylation sites, with GSK-3β capable of directly phosphorylating around 30 of these sites^5,6^. Hyperphosphorylated tau dissociates from microtubules and forms paired helical filaments and neurofibrillary tangles, characteristic lesions in Alzheimer’s disease. GSK-3β contributes to amyloid-β (Aβ) pathology by modulating the processing of amyloid precursor protein (APP) and stimulating the amyloidogenic pathway, which enhances Aβ peptide production and plaque formation. Aβ can activate GSK-3β, establishing a feedback loop that intensifies Aβ accumulation and tauopathy^7^. In addition to its role in plaques and tangles, GSK-3β is implicated in neuroinflammation and the activation of glial cells. Activated GSK-3β enhances microglial inflammatory signaling and cytokine production, contributing to chronic neuroinflammation in the Alzheimer’s disease brain^5,8^. Dysregulated GSK-3β adversely affects synaptic and neuronal integrity. Increased GSK-3β activity impairs synaptic plasticity and neurite development, while facilitating neuronal apoptosis. GSK-3β negatively affects long-term potentiation and synaptic strength, critical memory indicators. Its excessive activity has been demonstrated to cause neuronal death in Alzheimer’s disease models^8,9^.

Due to its pivotal involvement in various Alzheimer’s disease pathways, GSK-3β is regarded as a potential therapeutic target. Inhibition of GSK-3β may concurrently reduce tau hyperphosphorylation and Aβ production, potentially decelerating neurodegeneration^5,7^. Genetic or pharmacologic reduction of GSK-3β activity in Alzheimer’s disease models enhances cognitive performance and diminishes key pathologies. Several challenges complicate the development of drugs targeting GSK-3β. Selectivity presents a challenge due to the highly conserved nature of the ATP-binding site in GSK-3β among kinases, leading to small-molecule inhibitors frequently affecting off-target enzymes. Blood–brain barrier (BBB) penetration presents a significant challenge; numerous GSK-3β inhibitors demonstrating strong in-vitro potency do not achieve adequate concentrations in the brain. Safety is a concern due to the involvement of GSK-3β in various normal cellular functions, such as Wnt signaling and glucose regulation. Inhibition of GSK-3β can disrupt essential pathways and lead to toxicity^10^. Consequently, although GSK-3β represents a significant target, drug discovery must address challenges related to brain penetration, target specificity, and long-term safety.

**Fig. 1:**
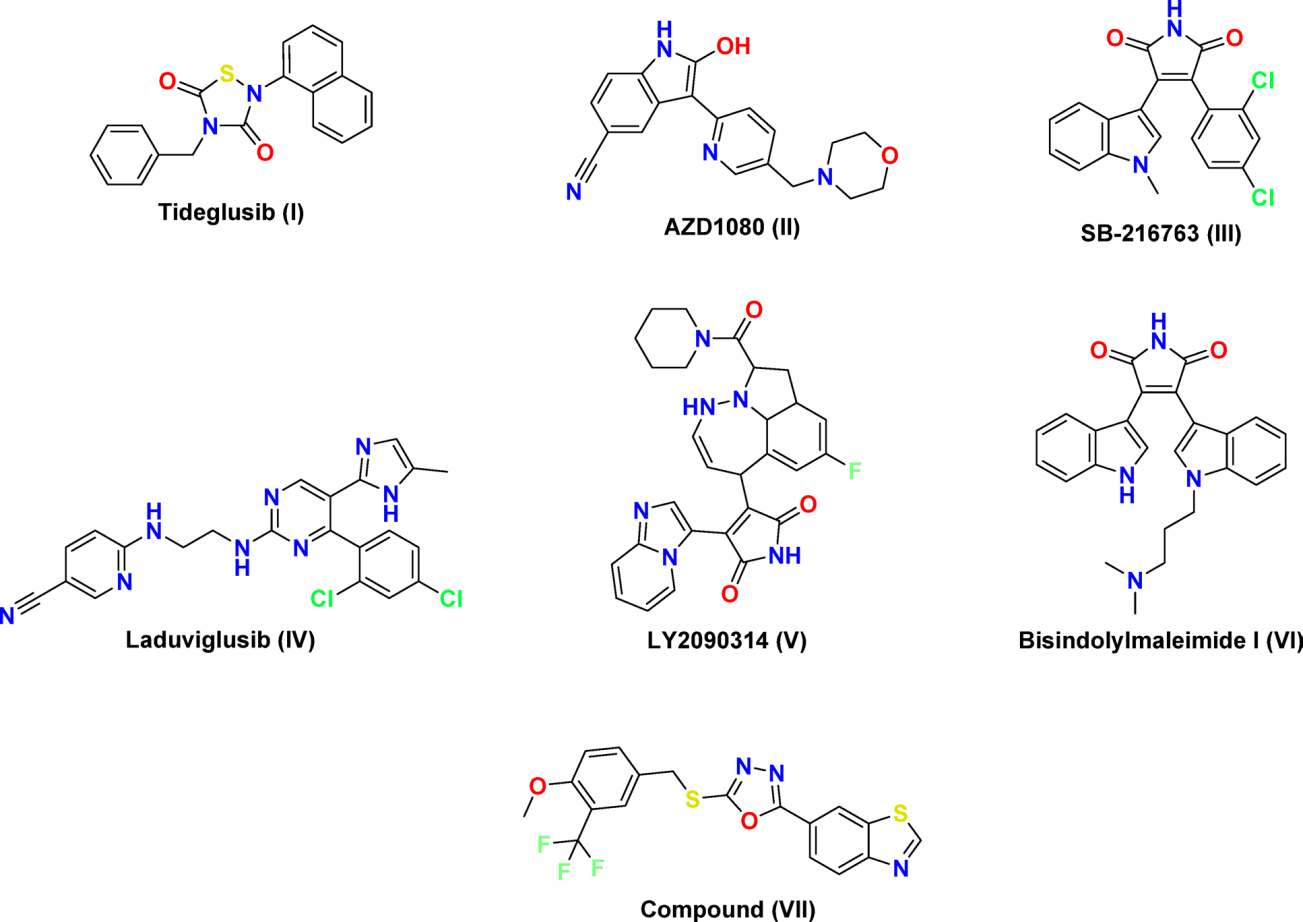
Chemical structures of seven reported GSK-3β inhibitors **(I-VII)**.

Despite these challenges, multiple GSK-3β-targeting agents have progressed to clinical or preclinical evaluation for Alzheimer’s disease or associated conditions (Fig. 1). **Tideglusib (I)** serves as an example of a non-ATP-competitive GSK-3β inhibitor. **Tideglusib (I)** demonstrated good tolerability in Phase I/II trials; however, it did not exhibit efficacy in 6-month trials for mild-to-moderate Alzheimer’s disease and in a trial for progressive supranuclear palsy^7^. **Lithium**, recognized for its mood-stabilizing properties and indirect inhibition of GSK-3β, has been subject to evaluation. Long-term studies indicate that chronic low-dose lithium may mitigate cognitive decline or hippocampal atrophy in Alzheimer’s disease and mild cognitive impairment; however, the findings are inconsistent and limited in scale^7^. Other selective GSK-3β inhibitors have been evaluated in humans or animals. **AZD1080 (II)** showed target engagement and decreased tau phosphorylation in rodents; however, its clinical development was halted due to toxicity concerns^9^. In preclinical models, several ATP-competitive inhibitors (e.g., **SB-216763 (III)**) and peptides have demonstrated efficacy in reducing Aβ-induced neurotoxicity, tau phosphorylation, and synaptic deficits; however, none have received approval for Alzheimer’s disease^11^. **Laduviglusib (IV)** is a potent, selective ATP-competitive inhibitor of GSK-3α/β, with an IC_50_ of approximately 6.7 nM for GSK-3β, effectively activating Wnt/β-catenin signaling in vitro. Despite its significant biochemical efficacy, **Laduviglusib (IV)** has not progressed beyond its status as a preclinical/research tool and lacks FDA approval as a therapeutic agent^12^. **LY2090314 (V)** is a highly effective GSK-3 inhibitor, with a reported IC_50_ of approximately 0.9 nM for GSK-3β. Although it has progressed to clinical testing, it has not obtained FDA approval for Alzheimer’s disease or other indications, despite its nanomolar potency and initial clinical evaluations^13^. **Bisindolylmaleimide I (VI)** is primarily recognised as a potent PKC inhibitor; however, it also demonstrates sub-micromolar inhibitory activity against GSK-3β in vitro, with reported IC_50_ values ranging from approximately 170 to 360 nM in lysate/immunoprecipitate assays. Despite this, it remains a research reagent and has not received FDA approval for clinical use as a GSK-3β inhibitor^14^. **Compound (VII)** is an inhibitor of GSK-3β, exhibiting an IC_50_ value of 3.1 nM. **Compound (VII)** shows potential for research in Alzheimer’s disease^15^.

Virtual screening has revolutionized drug discovery by saving time, money, and resources over high-throughput screening. Using experimental methods, researchers can test millions of chemicals in hours instead of months or years. Virtual screening reduces screening costs by orders of magnitude compared to conventional HTS, which can cost millions for major campaigns. Virtual screening can process thousands of compounds in hours, speeding up drug development and lead identification^16–19^.

These findings highlight the necessity for novel GSK-3β inhibitors that exhibit enhanced brain selectivity and safety profiles. Considering the target’s potential and associated challenges, in-silico methods are increasingly employed to identify and optimize novel GSK-3β inhibitors that may meet these criteria. To bridge the gap between static binding predictions and functional efficacy, modern computational workflows must integrate dynamic and pharmacokinetic evaluations^20^. Molecular dynamics (MD) simulations serve as a critical complementary tool to molecular docking, allowing for the rigorous assessment of protein-ligand complex stability, conformational shifts, and the persistence of key interactions over time^21^. Furthermore, the integration of in silico ADMET (Absorption, Distribution, Metabolism, Excretion, and Toxicity) profiling is paramount in early-stage CNS drug discovery. Because many potent in-vitro GSK-3β inhibitors fail clinically due to poor blood-brain barrier (BBB) penetration or off-target toxicity, predictive ADMET filtering ensures that only compounds with viable neuro-pharmacokinetic profiles and acceptable drug-likeness are prioritized for advancement^22^.

## Materials and methods

### Structure alignment and pharmacophoric modeling

Structure alignment and pharmacophore modelling were employed to identify and formalize the conserved interaction patterns necessary for GSK-3β inhibition and create ligand queries for subsequent virtual screening^23^. The structural alignment of representative inhibitors reveals common steric and electrostatic motifs, facilitating the extraction of a consensus geometry frequently linked to activity. Pharmacophore models generated from these conserved features encapsulate the critical chemical interactions and prioritize screening for compounds likely to replicate the observed binding mode^24^. The integration of ligand- and structure-based pharmacophore methodologies enhances the reliability of virtual hits by incorporating complementary data, specifically ligand similarities and detailed protein-ligand interaction geometries, ultimately leading to improved enrichment in subsequent screening efforts^25^.

The ligand-based model (**Pharmacophore model 1**) involved energy minimization of the promising GSK-3β inhibitors depicted in Fig. 1, followed by flexible structure alignment utilizing MOE v2019.0102 (Molecular Operating Environment (MOE), 2024.0601 Chemical Computing Group ULC, 910–1010 Sherbrooke St. W., Montreal, QC H3A 2R7, 2025). The optimal alignment was objectively determined based on three primary scoring criteria: maximizing the alignment uniformity (*U*) while minimizing both the electrostatic/van der Waals field score (*F*) and the steric shape overlap score (*S*) to achieve the highest consensus geometry. These seven reference inhibitors **(I-VII)** were strategically selected to encompass a broad chemical and pharmacological space. Structurally, they represent highly diverse chemotypes, ranging from thiadiazolidindiones to bisindolylmaleimides. Mechanistically, the set incorporates both ATP-competitive binders and non-ATP-competitive agents **(Tideglusib)**. To ensure pharmacological diversity, the compounds were selected across a broad spectrum of experimental in vitro potencies against GSK-3β: **LY2090314** (IC_50_ ≈ 0.9 nM), **Compound VII** (IC_50_ = 3.1 nM), **Laduviglusib** (IC_50_ ≈ 6.7 nM), **AZD1080** (IC_50_ = 31 nM), **SB-216,763** (IC_50_ = 34.3 nM), **Tideglusib** (IC_50_ = 60 nM), and **Bisindolylmaleimide I** (IC_50_ ≈ 170–360 nM). Utilizing this structurally and pharmacologically heterogeneous training set prevents the model from overfitting to the biases of a single chemical series. Instead, it ensures the extraction of a consensus geometry that captures only the fundamental spatial features strictly essential for GSK-3β inhibition, thereby maximizing the model’s generalizability and its capacity for scaffold-hopping during virtual screening. A consensus pharmacophore hypothesis was derived from the resulting superposition by identifying recurring features, including hydrogen-bond donors and acceptors, hydrophobic/aromatic centers, and relevant ionizable groups. The feature selection process strictly prioritized chemical moieties that were universally conserved across the diverse training set. By doing so, the final hypothesis captured only the essential 3D pharmacophoric determinants required for target engagement, actively filtering out scaffold-specific artifacts. Feature positions and radii were assigned to represent the observed geometry, and excluded volumes were incorporated where steric data indicated possible clashes. The model was optimized to enhance the mapping of known actives while reducing matches with decoys, and it was subsequently exported as a query file for virtual screening applications.

The structure-based model (**Pharmacophore model 2**) utilized the crystal structure with **PDB code 3GB2**, which was uploaded to the Pharmit webserver^26^. This specific crystal structure was selected due to its excellent resolution and the presence of a well-defined co-crystallized inhibitor in the active site, providing a highly reliable structural template for the ATP-binding pocket. The co-crystallized ligand served as the feature source for the automatic detection and proposal of pharmacophore features. Features were manually reviewed and adjusted to maintain relevant hydrogen-bond donors and acceptors as well as hydrophobic and aromatic interactions. Specifically, the selection process prioritized and retained features that mapped directly to known critical interactions within the GSK-3β ATP-binding pocket, such as the essential hinge-binding contact with Lys85. Furthermore, protein-derived excluded volumes were enabled to avoid sterically infeasible hits.

### Ligand library and selection

A search of pharmacophore models was conducted against the ZINCPharmer database, which contains 21,777,093 compounds^27^. Each pharmacophore query was submitted individually, and the resulting hits were ranked based on fit/RMSD to the query. Compounds exhibiting a pharmacophore-mapping RMSD of ≤ 1.0 Å were considered promising matches. This specific threshold was selected to establish an optimal balance between geometric fidelity and chemical diversity. A more restrictive cut-off (≤ 0.5 Å) would heavily penalize minor spatial deviations, thereby severely limiting scaffold hopping and potentially excluding novel, active chemotypes, a critical consideration given that the structure-based query yielded a highly focused set of only 36 candidates. Conversely, employing a more permissive cut-off (≥ 1.5 Å) would notably increase the false-positive rate by retrieving molecules that deviate too far from the ideal binding geometry. This would introduce excessive structural noise and create an unmanageable computational burden for the subsequent in-silico docking and molecular dynamics evaluations. Thus, the 1.0 Å limit ensures the spatial conservation of essential interacting features while maintaining a sufficiently broad chemical space for robust hit discovery. Retrieved hits, including ZINC IDs and SMILES, were exported for further processing and utilized in subsequent docking and scoring analyses.

### Molecular docking

Molecular docking of the retained hits was employed to assess binding affinities and to compare predicted scores with the co-crystallized ligand, which has a binding energy of -6.8 kcal/mol. Ligands were prepared utilizing OpenBabel^28^, which involved converting to 3D, adding polar hydrogens, determining appropriate protonation states, minimizing where applicable, and converting to the docking format. The receptor (**PDB ID: 3GB2**) was prepared by eliminating crystallographic waters and non-protein molecules, adding polar hydrogens, and converting to PDBQT file format. Docking was conducted using AutoDock Vina v1.1.2.^29^, employing a grid box centered at coordinates *x = 33.177*,* y = 14.194*,* z = 2.494*. The box dimensions were *55 × 55 × 55*, with a grid spacing of 0.375 Å, and the search exhaustiveness was set to 32. Ligand affinities were evaluated using the standard AutoDock Vina empirical scoring function, which approximates the binding free energy (in kcal/mol) by calculating the sum of distance-dependent intermolecular and intramolecular steric and thermodynamic interactions. Prior to screening, the docking protocol was rigorously validated by redocking the native co-crystallized ligand into the active site. While AutoDock Vina generated multiple alternative binding poses, the top-ranked pose (lowest binding energy) successfully reproduced the experimental crystallographic conformation with an RMSD of 1.45 Å, confirming the reliability of the grid parameters. Consequently, for all screened virtual hits, the pose with the lowest predicted binding energy was selected for ranking and visual inspection of ligand-protein interactions. The comprehensive 2D interaction analysis of these top-ranked AutoDock Vina poses, mapping specific hydrogen bonds and π-based electrostatic contacts, was generated using BIOVIA Discovery Studio Visualizer. The dynamic structural persistence and overall binding stability of these selected static-in-silico poses were subsequently ensured and verified through 100-ns molecular dynamics (MD) simulations.

### ADME studies

Top docking hits, those with a predicted binding affinity more favorable than the co-crystallized ligand, were submitted to the SwissADME webserver in canonical SMILES format to assess drug-likeness and brain penetration^30^. To ensure rigorous and reproducible triage, strict exclusion criteria were established upfront: any compound exhibiting more than one violation of Lipinski’s Rule-of-Five was categorized as non-compliant and immediately excluded from further analysis. We documented each compound’s Lipinski Rule-of-Five descriptors (Molecular Weight, cLogP, H-bond donors/acceptors) and proceeded only with those meeting this threshold. The BBB and gastrointestinal absorption permeation were evaluated using the prediction outputs from SwissADME, specifically the BOILED-Egg model and associated descriptors. Compounds identified as permeant to the BBB were marked for further investigation. An additional criterion was applied for hits derived from Pharmacophore model 1: compounds needed to be classified by SwissADME solubility estimates (ESOL/SILICOS-IT) as at least “moderately soluble.” All SwissADME result files, including Lipinski parameters, BOILED-Egg classification, solubility class, and other relevant descriptors, were exported to prioritize compounds for subsequent experimental validation.

### In-silico toxicity prediction

In-silico toxicity predictions were conducted using the ProTox-3.0 webserver to assess the safety profiles and potential developmental liabilities of the prioritized hits^31^. The canonical SMILES of the leading candidates **(SB1** and **LB1)** were submitted to predict their acute oral toxicity, quantified as the median lethal dose (LD_50_ in mg/kg), and to classify them into standard Toxicity Classes, which range from Class 1 (fatal) to Class 6 (non-toxic). The server’s machine-learning models were employed to identify specific organ toxicities (e.g., hepatotoxicity, nephrotoxicity) and toxicological endpoints (e.g., mutagenicity, immunotoxicity) to provide early structural warnings before in-vitro testing^32^.

### Molecular dynamics

Molecular dynamics simulations were conducted using GPU-accelerated GROMACS 2025.3 to analyze the chosen protein-ligand complexes^33^. Protein coordinates and topologies were generated using pdb2gmx with the CHARMM27 force field, while ligand topologies for the candidate hits and the co-crystallized ligand were created through the SwissParam web server^34^. The protein and ligand topology files were subsequently integrated to generate the final complex input files. Each complex was situated in a cubic simulation box, ensuring a minimum clearance of 1.0 nm between the protein and the box boundary. The system was solvated using the TIP3P water model and neutralized by substituting solvent molecules with suitable counter-ions^35,36^. Steric clashes were alleviated through energy minimization using steepest descent and conjugate gradient algorithms. During all subsequent dynamic steps, bonds involving hydrogen atoms were constrained using the LINCS algorithm. Short-range van der Waals interactions were evaluated using a 1.2 nm cutoff with a force-switch modifier applied from 1.0 nm. Long-range electrostatics were treated using the Particle Mesh Ewald (PME) method with a 1.2 nm cutoff and a 0.16 nm grid spacing. Systems underwent equilibration in NVT and NPT, each lasting 10 ns with a 2-fs integration time step. The system temperature was maintained at 300 K using the modified Berendsen (V-rescale) thermostat. During the NPT equilibration, pressure was maintained at 1.0 bar using the Berendsen barostat. Production trajectories of 100-ns were subsequently recorded under NPT conditions using the Parrinello-Rahman barostat to analyze dynamic behavior.

### Retrospective validation of pharmacophore models using ROC curve analysis

The discriminatory ability of the ligand-based and structure-based pharmacophore models was evaluated by retrospective receiver operating characteristic (ROC) curve analysis using active-decoy validation datasets. Experimentally confirmed GSK-3β active compounds were used as the positive set, while property-matched decoys were generated using LUDe at a ratio of 50 decoys per active compound. For the ligand-based pharmacophore model, 3 active compounds and 150 LUDe-generated decoys were used. For the structure-based pharmacophore model, 1 active compound and 50 LUDe-generated decoys were used. The generated decoys were selected to possess similar physicochemical properties to the active compounds while maintaining structural dissimilarity, thereby allowing assessment of the ability of each pharmacophore model to distinguish true actives from presumed inactive compounds. The active-decoy datasets were imported into MOE and screened against their corresponding pharmacophore models using the pharmacophore Search protocol. For each model, compounds were ranked according to their pharmacophore RMSD value, where lower RMSD values indicated better fitting to the pharmacophoric features. Only the best-matching conformation per molecule was considered for analysis. Non-retrieved compounds were assigned a poor RMSD value of 999 to place them at the bottom of the ranked dataset.

For ROC curve construction, RMSD values were transformed into negative RMSD scores, since ROC analysis requires higher scores to indicate better predicted activity. Therefore, compounds with lower RMSD values had higher ranking scores. The final datasets were analyzed using GraphPad Prism 10.1.2 Demo (GraphPad Software, San Diego, CA, USA). to generate ROC curves and calculate the area under the curve (AUC), sensitivity, specificity, confidence intervals, and *p* values.

## Results

### Structure alignment and pharmacophoric models

**Fig. 2:**
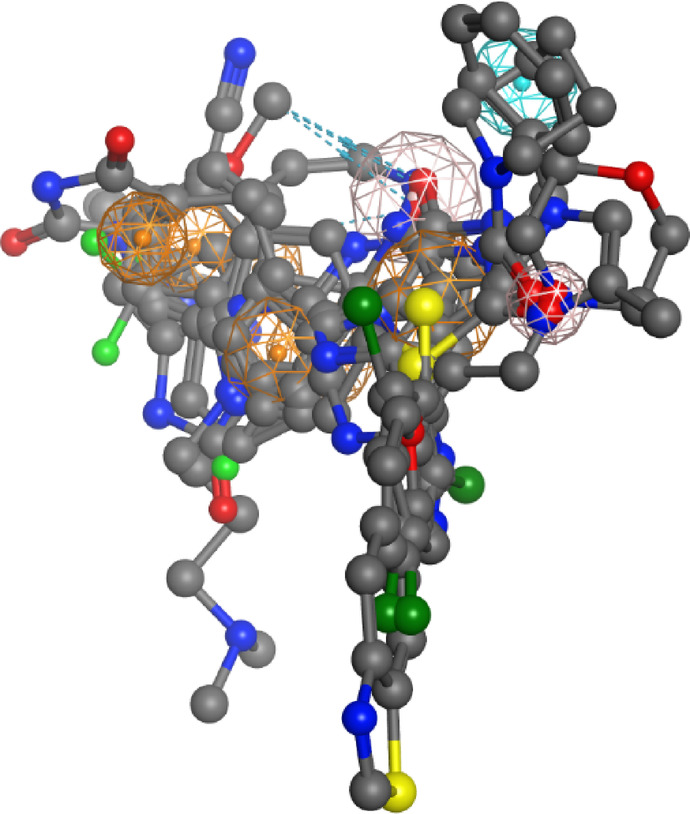
Structure alignment of seven representative GSK-3β inhibitors **(I-VII)** and derived pharmacophore model.

In the flexible structure alignment of the seven selected GSK-3β inhibitors **(I–VII)**, the optimal scoring pose yielded U = 41.8961, F = -108.0886, and S = -66.1924, as illustrated in Fig. 2. Our analysis indicates that these metrics encapsulate complementary aspects of the superposition. U represents the alignment and uniformity across the ligand set, with higher values indicating a more consistent standard frame of reference^37^. F is a field score that measures the electrostatic and van der Waals field overlap between the superposed molecules, where more negative values denote better field agreement^38^. S quantifies steric complementarity or shape overlap, with more negative values indicating an improved steric fit^39^. The combination of a high U with strongly negative F and S values suggests a well-conserved arrangement of key pharmacophoric elements among the seven inhibitors **(I-VII)**, establishing a solid foundation for ligand-based pharmacophore extraction.

**Fig. 3:**
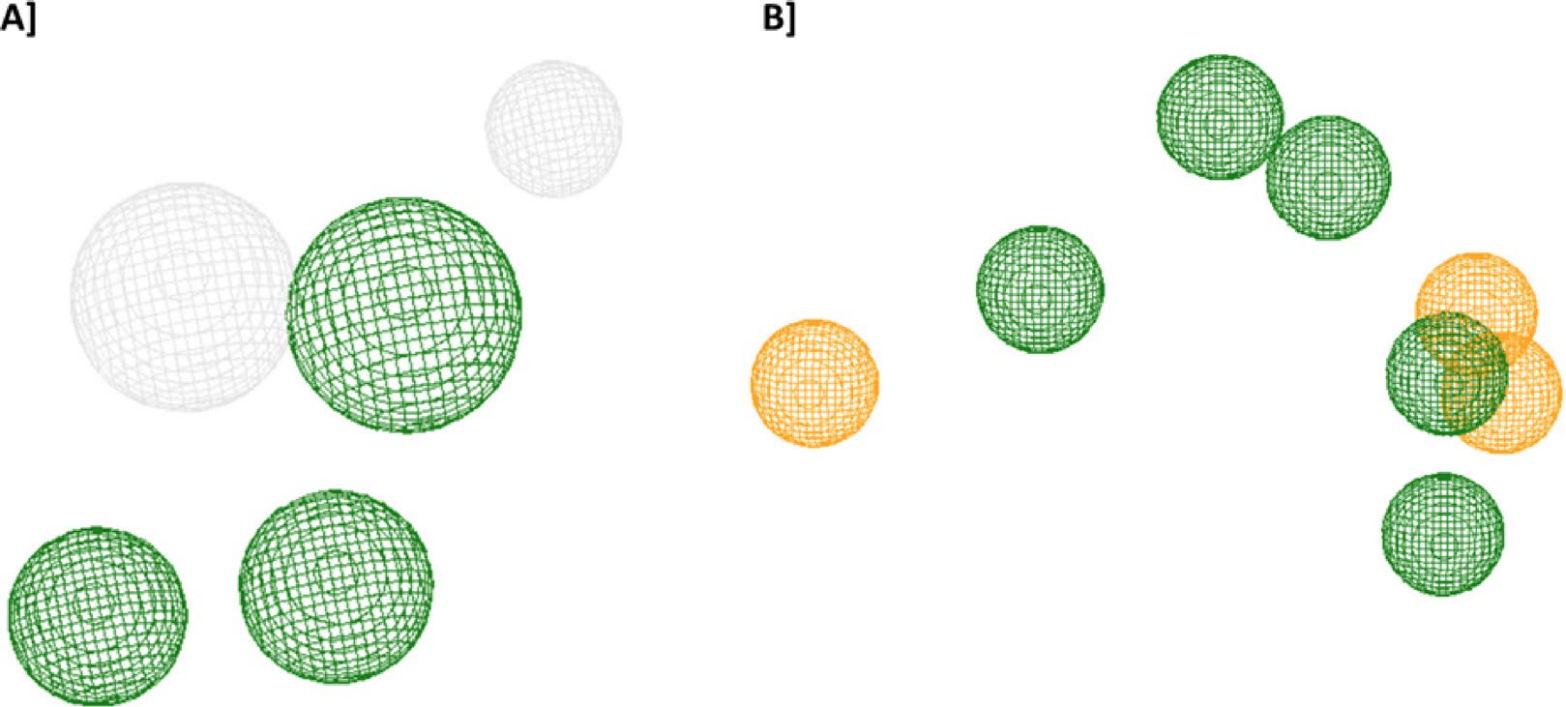
Visual representation and 3D spatial arrangement of the generated pharmacophore features used for virtual screening. (**A**) The ligand-based consensus model and (**B**) the structure-based model derived from **PDB 3GB2**. The feature colors indicate the corresponding pharmacophoric elements in the displayed model [green = hydrophobic, orange = hydrogen bond acceptor, gray or white = overlapping aromatic or excluded volume features].

A pharmacophore based on ligands was constructed from the alignment by identifying recurring aromatic, hydrophobic, and hydrogen-bonding features in the superposed compounds. Several feature types were co-localized: an aromatic and hydrophobic pair at (0.62, 1.85, -0.75), another aromatic/hydrophobic pair at (-3.18, -1.04, 0.98), and a third aromatic/hydrophobic pair at (-0.66, -1.04, -0.05). Two distinct pairs of hydrogen-bond acceptor/donor features were identified at (2.61, 3.60, -0.91) and (-1.67, 2.48, -0.79), illustrating their 3D spatial arrangement as shown in Fig. 3A. The .ph4 pharmacophore query was applied to the ZINCPharmer collection, yielding 1,085 hits that met our fitting criteria.

A structure-based pharmacophore was generated from the GSK-3β crystal structure (**PDB ID: 3GB2**) using the Pharmit webserver, deriving features from the co-crystallized ligand. The model consisted of three hydrogen-bond acceptor features positioned at approximately (36.17, 14.51, -0.11), (35.74, 15.83, 0.02), and (25.26, 15.31, 4.45), along with five hydrophobic points clustered around (28.76, 16.72, 3.67), (33.28, 18.26, 1.46), (31.55, 19.32, 2.23), (35.27, 14.92, 0.54), and (35.23, 12.32, 0.95), as shown in Fig. 3B. The pharmacophore was exported as a .json query and screened in ZINCPharmer, resulting in 36 candidate hits. Only compounds aligned with the pharmacophore with an RMSD ≤ 1.0 Å were selected for further docking and ADME evaluation in ligand- and structure-based searches.

### Initial molecular docking filtering

Molecular docking is essential in structure-based drug discovery, as it predicts the orientation and interaction of small molecules within a protein’s binding site^40,41^. In our hierarchical virtual screening pipeline, hits were sequentially filtered based on geometric pharmacophore mapping (RMSD ≤ 1.0 Å), predicted binding affinity, and strict ADME/developability thresholds. It offers a rapid and cost-effective approach to prioritizing extensive compound libraries for experimental evaluation. Docking algorithms sample ligand conformations and poses, evaluating them with scoring functions that approximate binding affinity. This process enables researchers to rank hits from virtual screens, identify key protein-ligand contacts such as hydrogen bonds, hydrophobic pockets, and π-π interactions, and generate hypotheses for structure–activity relationships and lead optimization^42–44^. Molecular docking was conducted for all compounds obtained from the ligand- and structure-based pharmacophore screens, using the energy of the co-crystallized ligand (-6.8 kcal/mol) as a benchmark for filtering. Of the 1,085 hits identified by the ligand-based pharmacophore, 957 compounds (approximately 88.2%) demonstrated predicted binding affinities exceeding − 6.8 kcal/mol and were consequently retained. Of the 36 candidates identified through the structure-based pharmacophore, 22 compounds (approximately 61.1%) exhibited superior scores to the co-crystallized ligand and were subsequently advanced.

### ADME filtering

In-silico ADME prediction was conducted to efficiently evaluate the drug-likeness and developability of virtual hits before experimental testing. Computational ADME tools assess essential properties that affect absorption, distribution, metabolism, and excretion^45^. We prioritized BBB permeability and Lipinski compliance due to compounds needing to achieve therapeutically relevant concentrations in the central nervous system for our target indication, Alzheimer’s disease^46^. As an explicit exclusion criterion, compounds presenting more than one Lipinski violation were strictly rejected to minimize developability concerns. SwissADME was utilized to predict BBB permeation and associated properties, including P-glycoprotein efflux propensity^47^ and polar surface area, allowing for the deprioritization of potent docking hits that do not possess the necessary physicochemical profile for brain exposure^48^. For the ligand-based pharmacophore hits, we established an additional criterion of at least “moderate” aqueous solubility^49^. This requirement is essential as sufficient solubility facilitates systemic exposure, allows for reliable in-vitro testing and formulation, and ensures that a compound’s lipophilicity-driven blood-brain barrier potential is not compromised by inadequate dissolution. Collectively, these ADME filters aid in balancing predicted potency with a feasible opportunity for advancing a CNS-active candidate.

**Fig. 4:**
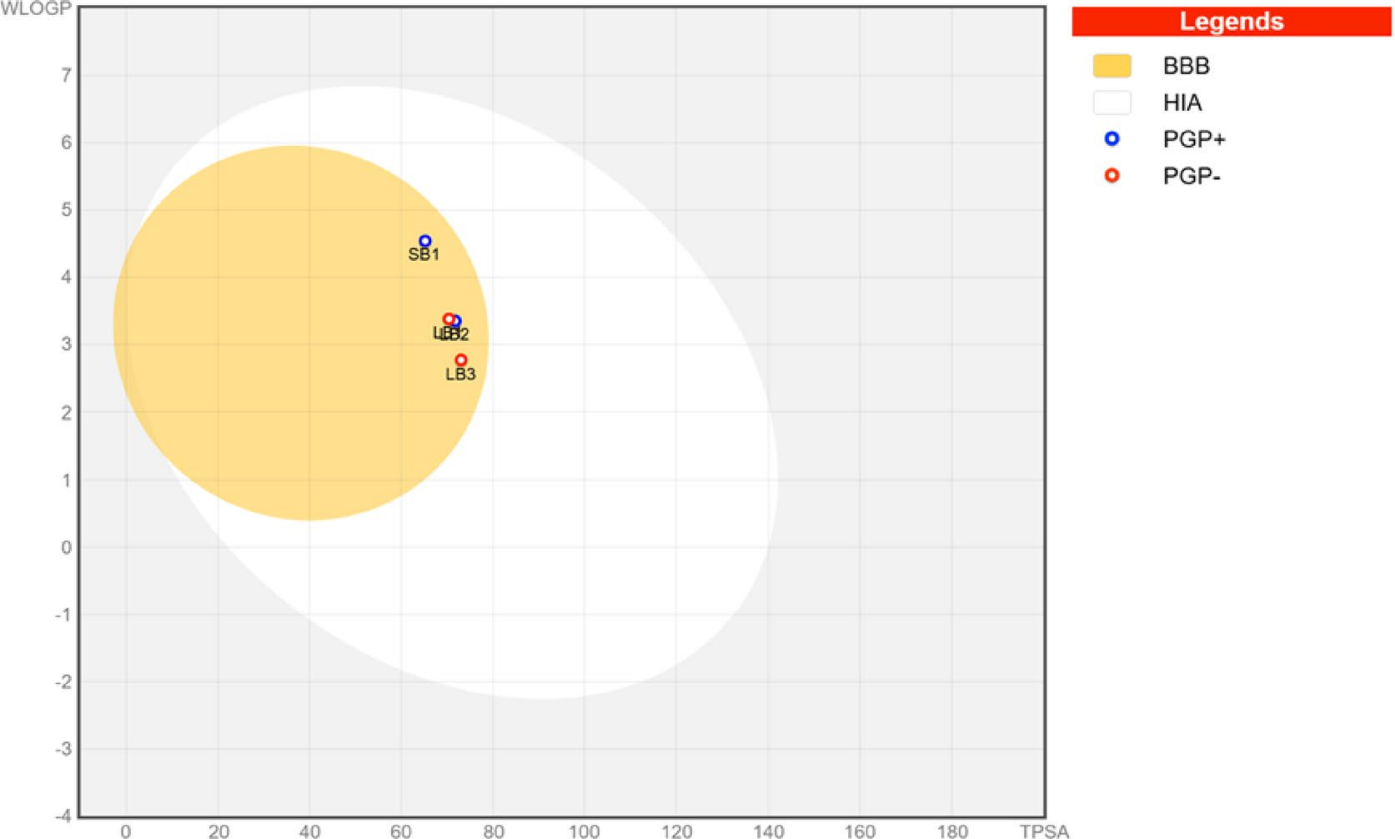
SwissADME BOILED-Egg model predictions for prioritized GSK-3β inhibitor hits.

All ligand-based hits **(LB1-LB3)** satisfy the “moderately soluble” ESOL classification, serving as an additional filter for the ligand-based pharmacophore series (Table 1). In contrast, the structure-based hit **(SB1)** is predicted to be poorly soluble by Silicos-IT, despite being classified as “moderately soluble” by ESOL, indicating a potential concern for the developability of **(SB1)**. All compounds show high predicted gastrointestinal absorption and are predicted to be BBB-permeant, which is desirable for a CNS target such as GSK-3β in Alzheimer’s disease. Two compounds are predicted to be P-glycoprotein substrates **(SB1** and **LB2)**, which could limit brain exposure despite predicted BBB permeation and thus should be considered when prioritizing compounds (Fig. 4). All compounds adhere to Lipinski’s Rule-of-Five with no violations and exhibit identical predicted oral bioavailability scores of 0.55. Synthetic accessibility scores vary from approximately 2.2, indicating ease of synthesis, to 3.7, representing greater difficulty, with PAINS alerts recorded as zero throughout the series.

**Table 1:** Predicted physicochemical and ADME properties of prioritized hits from the structure- and ligand-based pharmacophore screens, as computed by SwissADME.

ID	MW (g/mol)	LogP	TPSA (Å²)	BBB permeant	ESOL solubility class	*P*-gp substrate
SB1	353.48	3.89	65.24	Yes	Moderately soluble	Yes
LB1	293.32	2.98	70.42	Yes	Moderately soluble	No
LB2	295.29	2.85	71.81	Yes	Moderately soluble	Yes
LB3	296.30	2.80	73.06	Yes	Moderately soluble	No

### In-silico toxicity profiling

**Fig. 5:**
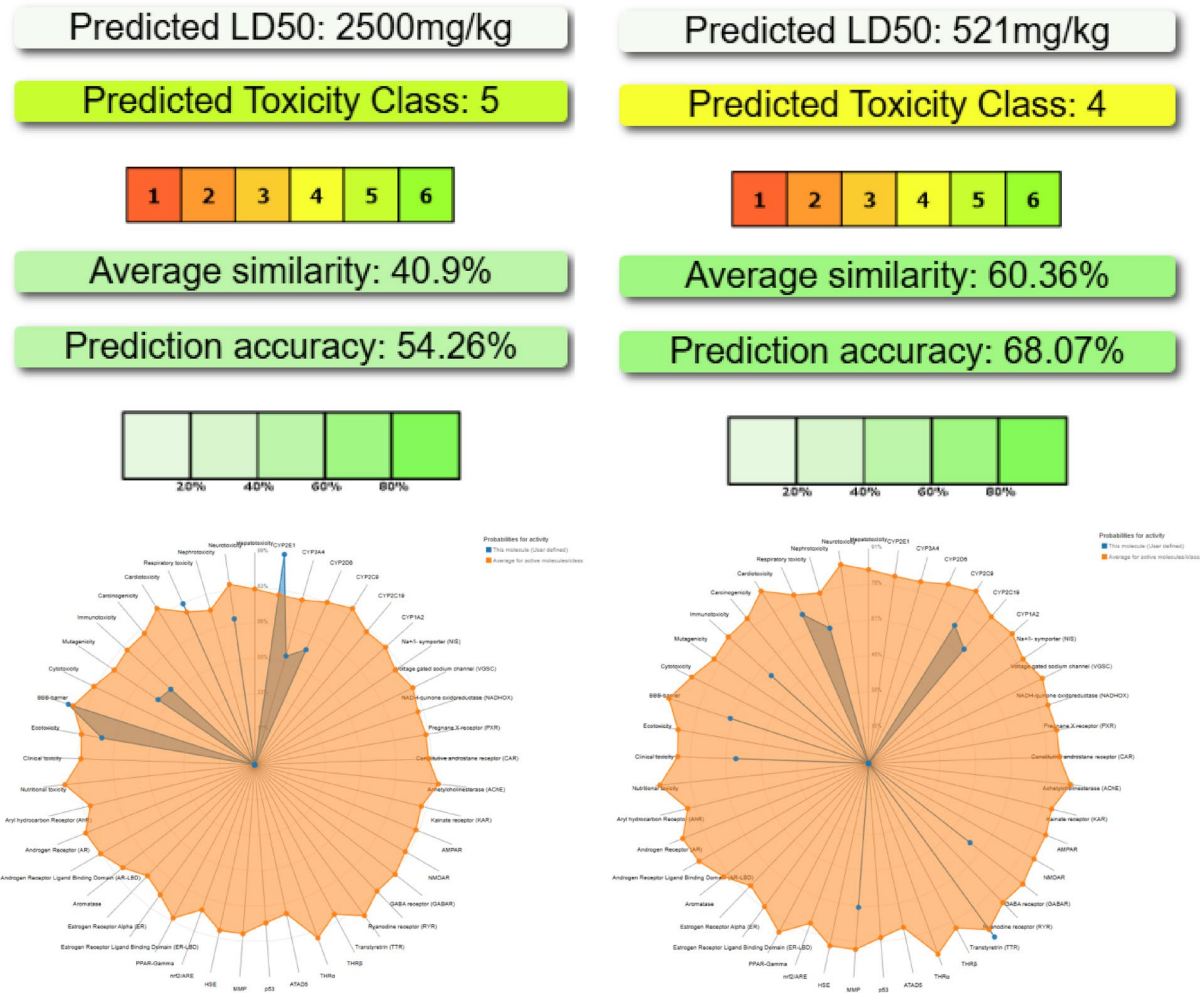
In-silico toxicity profiling and endpoint prediction of the top prioritized GSK-3β candidates. The figure illustrates the predicted acute oral toxicity (LD_50_), designated toxicity class, prediction accuracy, and radar plots detailing specific organ and endpoint liabilities for the structure-based hit **SB1** (**left**) and the ligand-based hit **LB1** (**right**).

Following the ADME evaluation, the safety profiles of the top candidates, **SB1** and **LB1**, were assessed using the ProTox-3.0 webserver. The predicted acute oral toxicity and specific endpoint liabilities provided critical insights into the developability of these compounds. **SB1** demonstrated a highly favorable acute toxicity profile, with a predicted LD_50_ of 2500 mg/kg, placing it in Toxicity Class 5 (indicating it is generally considered to have low acute toxicity). However, specific endpoint predictions highlighted potential risks for respiratory toxicity (probability: 0.82) alongside mild alerts for neurotoxicity and mutagenicity, as illustrated in Fig. 5.

In contrast, **LB1** exhibited a predicted LD_50_ of 521 mg/kg, classifying it into Toxicity Class 4 (harmful if swallowed). This indicates a moderate acute toxicity profile compared to **SB1**. The endpoint analysis for LB1 showed potential liabilities for respiratory toxicity (probability: 0.69) and nephrotoxicity (probability: 0.59).

Notably, both compounds yielded “Active” predictions for blood-brain barrier (BBB) permeability within the ProTox-3.0 screening. Rather than a liability, this corroborates our SwissADME findings and confirms a crucial pharmacokinetic prerequisite for Alzheimer’s disease therapeutics. While these in-silico toxicity alerts serve as a valuable early-warning system to guide future structural optimization, they are strictly predictive and necessitate rigorous in vitro and in vivo toxicological validation during subsequent lead optimization phases.

### Molecular docking of final hits for interaction analysis

**Fig. 6:**
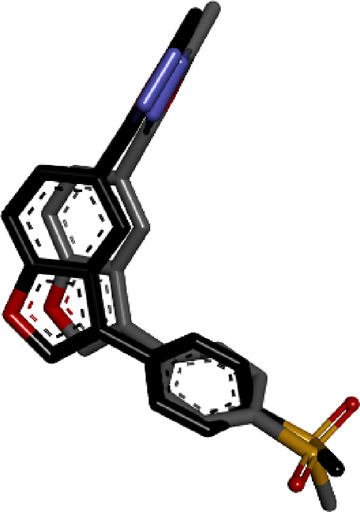
Validation of the docking protocol.

The docking protocol was validated by re-docking the co-crystallized ligand into the binding pocket of GSK-3β (**PDB ID: 3GB2**). The predicted binding pose achieved a root-mean-square deviation (RMSD) of 1.45 Å compared with the experimental crystallographic conformation, indicating good reliability of the docking setup and justifying its use for subsequent analyses (Fig. 6).

The docking scores of the six prioritized hits were compared with that of the co-crystallized ligand (-6.80 kcal/mol) to evaluate their predicted binding poses and relative scoring metrics toward GSK-3β. All candidate molecules demonstrated more favorable predicted binding affinities than the reference ligand. However, it is important to note that these docking scores are semi-quantitative estimates used for compound prioritization; more negative scores do not inherently guarantee proportionally higher in-vitro inhibitory potency. The structure-based hit **SB1** yielded a binding affinity of -7.16 kcal/mol, while the ligand-based hits **LB1**, **LB2**, and **LB3** showed values of -7.28, -7.00, and − 7.39 kcal/mol, respectively (Table 2). These results suggest that all four hits bind more tightly than the crystallographic reference, with **LB1** and **LB3** emerging as the most promising candidates for further structural and dynamic investigation.

**Table 2:** Binding affinities of the structure-based hit **(SB1)**, ligand-based hits **(LB1-LB3)**, and the co-crystallized ligand within the active site of GSK-3β.

ID	Structure	Binding affinity (kcal/mol)
Co-crystallized (PDB: 3GB2)		-6.80
SB1		-7.16
LB1		-7.28
LB2		-7.00
LB3		-7.39

**Fig. 7:**
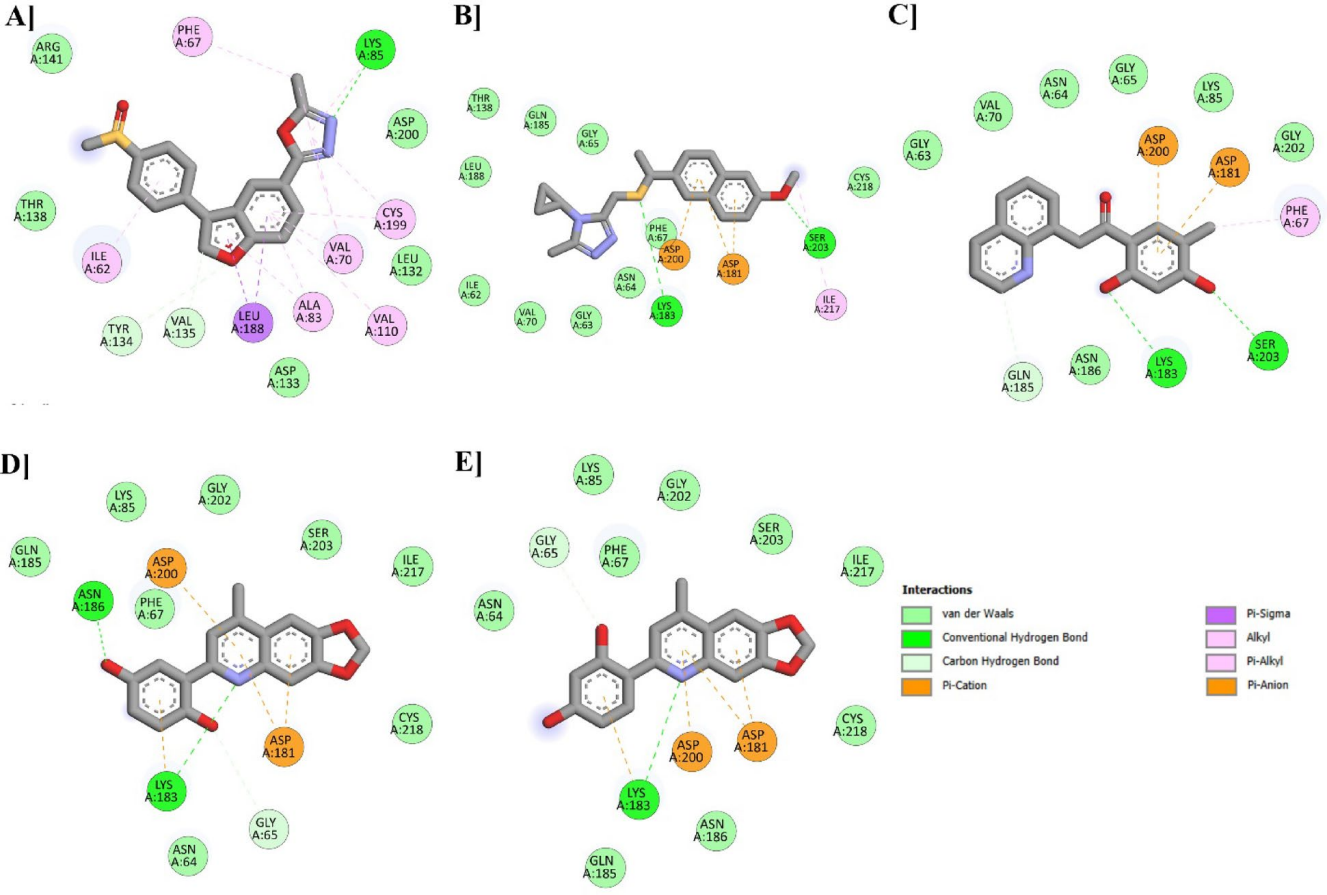
Two-dimensional interaction diagrams of the **co-crystallized ligand** (**A**), **SB1** (**B**), **LB1** (**C**), **LB2** (**D**), and **LB3** (**E**) docked within the active site of GSK-3β (**PDB ID: 3GB2**).

The **co-crystallized** ligand of GSK-3β formed a single hydrogen bond between its oxadiazole scaffold and Lys85, along with multiple hydrophobic contacts involving Ile62, Phe67, Val70, Ala83, Lys85, Val110, Leu188, and Cys199 (Fig. 7). In contrast, the newly identified hits established richer interaction profiles. The structure-based hit **SB1** formed two hydrogen bonds, one between its sulfur atom and Lys183 and another between its oxygen atom and Ser203, in addition to π-anion interactions between its naphthyl group and Asp181/Asp200. Similarly, **LB1** reproduced the Lys183 and Ser203 hydrogen bonds *via* its dihydroxy-substituted phenyl ring, accompanied by π-anion interactions with Asp181 and Asp200. **LB2** also maintained the Lys183 hydrogen bond and introduced an additional interaction with Asn186; its extended conjugated system enabled both π-anion and π-cation interactions. **LB3** showed an interaction profile comparable to **LB2** but lacked the Asn186 hydrogen bond.

These additional hydrogen bonds and π-based interactions are predicted to enhance affinity and specificity toward GSK-3β. Hydrogen bonding with residues such as Lys183, Asn186, and Ser203 likely improves binding stability by anchoring the ligands within the active site. In contrast, π-anion and π-cation interactions with Asp181, Asp200, and positively charged residues further reinforce electrostatic complementarity. These enriched interaction profiles suggest that the identified hits establish robust structural contacts with GSK-3β, yielding highly favorable computational scoring metrics. While these predictive profiles are encouraging, subsequent in-vitro enzymatic assays are strictly required to translate these in-silico docking scores into confirmed inhibitory potency.

### MD stability and interactions

Molecular dynamics (MD) gives biomolecular systems a time-resolved, physics-based picture that static crystal structures cannot. MD shows whether a protein retains its folded architecture or undergoes functionally important rearrangements, whether ligands hold their binding posture or diffuse and reorient, and on what time frames these events occur by propagating atom positions under realistic force-field stresses^50^. Before doing more expensive studies, this temporal information connects structural snapshots to thermodynamic and kinetic behavior to inform theories about binding affinity, selectivity, and hit stability^51,52^.

In MD analysis, root-mean-square deviation (RMSD) is basic but powerful. After aligning the protein, ligand RMSD measures ligand binding strength, while backbone RMSD records global protein structural drift and checks system equilibration immediately^53^. Low and tightly distributed backbone RMSD values indicate the protein retains its crystallographic fold and the complex does not experience significant, artifactual rearrangements; low ligand RMSD values indicate pose retention and fewer major ligand translations or rotations inside the binding pocket^54^. Two RMSD measurements assess simulation fidelity and binding stability across ligands or pharmacophore-derived models^55,56^.

**Fig. 8:**
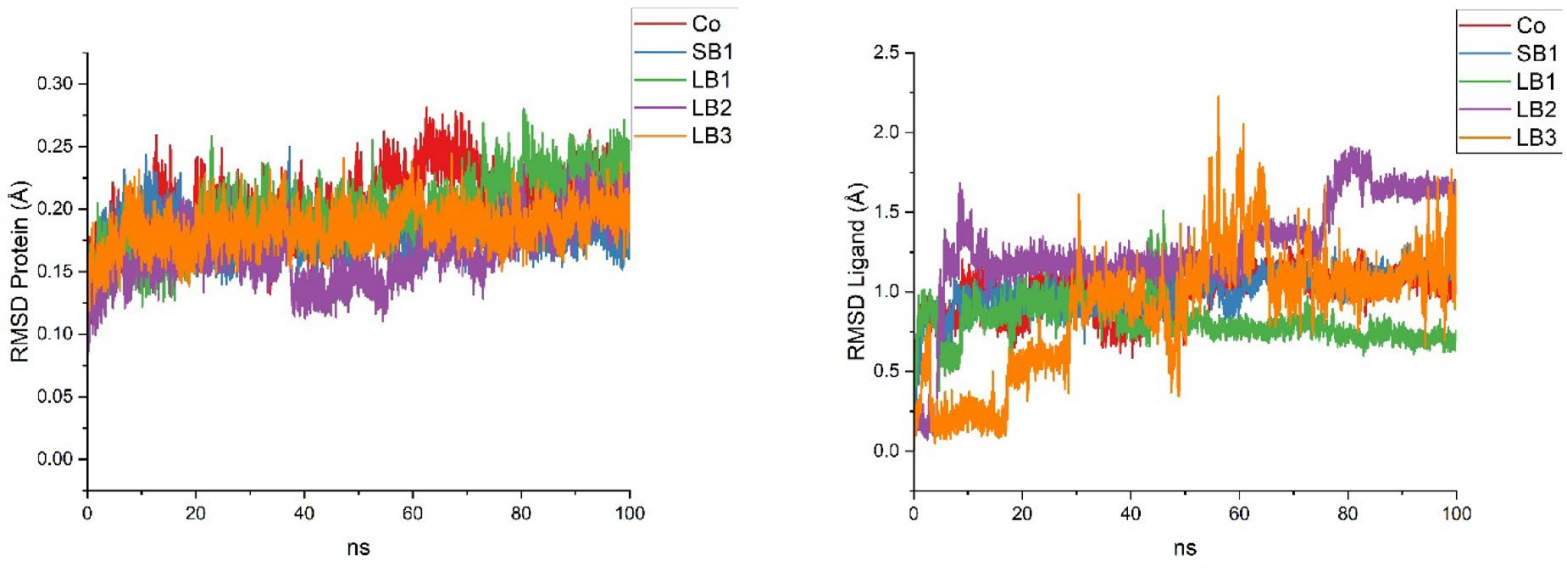
RMSD profiles for five 100-ns MD runs on GSK-3β, showing backbone stability **(left panel)** and ligand positional fluctuations **(right panel)**.

The 100-ns simulations indicate notable variations between the co-crystallized ligand (**Co**), the structure-based pharmacophore model **SB1**, and the ligand-based model **LB1** (Fig. 8). **SB1** has the lowest protein perturbation (backbone RMSD ≈ 0.180 Å), compared to the co-crystallized ligand (**Co**: ≈ 0.202 Å) and slightly lower than **LB1** (≈ 0.197 Å) in the full 0-100 ns traces. This suggests that the protein accommodates **SB1** with less global drift. The ligand RMSD of **LB1** is ≈ 0.815 Å, considerably lower than the co-crystallized ligand (**Co**: ≈ 1.000 Å) and slightly lower than **SB1** (∼ 0.997 Å) along the trajectory, indicating a tighter binding site retention.

In the final 20 ns of the production-like interval (80–100 ns), **SB1** has a lower and more tightly distributed backbone RMSD than the co-crystallized ligand (**Co**: ≈ 0.216 Å) and **LB1** (≈ 0.231 Å), indicating less global structural perturbation in the equilibrated complex. During the production phase, **LB1** remains the most conformationally constrained ligand in the binding site, with a ligand RMSD of 0.721 Å, compared to the co-crystallized ligand at 1.081 Å and **SB1** at 1.115 Å. More insight comes from predicted stabilization timeframes. The rolling-mean stability test shows that **SB1**’s backbone stabilizes early (28.7 ns) and remains narrowly distributed throughout the simulation, while the co-crystallized ligand and **LB1** stabilize later (**Co** ~ 74.7 ns and **LB1** ~ 76.6 ns). For ligand RMSD, the co-crystallized ligand stabilizes at 73.7 ns, **LB1** at 78.6 ns, and **SB1** at ≈ 86.0 ns, indicating that **SB1** has a rapidly equilibrated protein backbone. Still, its ligand requires longer rearrangements to stabilize.

The root-mean-square fluctuation (RMSF) is a key indicator for ligand-induced stabilization because it gives residue- or frame-resolved information about a protein’s local flexibility during MD simulations^57^. RMSF complements RMSD by showing which protein regions (or the whole-protein average, when reported as a single curve) become more rigid or flexible with different ligands. Reduced RMSF in ligand-bound simulations often indicates stronger, more persistent contacts and/or a reduction in local conformational sampling, which can favor high-affinity binding. RMSF comparisons in GSK-3β reveal how new pharmacophore-derived molecules impact backbone and side-chain mobility compared to the crystallographic reference^58,59^.

**Fig. 9:**
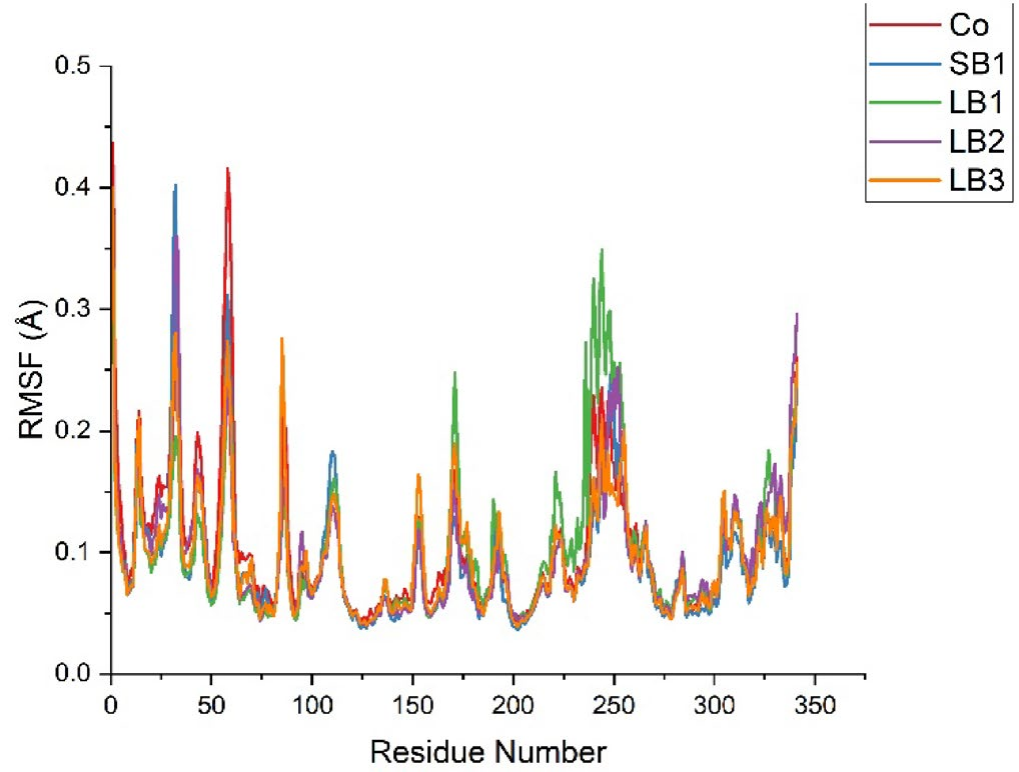
RMSF values for the five trajectories for the 100-ns production run.

The average RMSF values for all simulated frames were: **Co** = 0.1051 Å, **SB1** = 0.0927 Å, and **LB1** = 0.1022 Å (Fig. 9). Compared to the co-crystallized ligand, **SB1** decreases GSK-3β average fluctuation by ≈ 11.8%, while **LB1** only reduces the mean by ≈ 2.8%. Comparing solely equilibrated segments (frames from stable plateau start to end), the mean RMSF values are **Co** = 0.0948 Å, **SB1** = 0.0867 Å, and **LB1** = 0.1003 Å. **SB1** has a lower RMSF than **Co** (≈ 8.6% lower) in the equilibrated regime, but **LB1** has a slightly higher mean (≈ 5.8% higher). Maximum instantaneous fluctuations (peaks) are **Co**_max_ = 0.4371, **SB1**_max_ = 0.4020, and **LB1**_max_ = 0.3491. **Co** showed 22 large spikes (RMSF > 0.20 Å), **SB1** 14, and **LB1** 25, showing that **SB1** exhibits lower average fluctuations and fewer large intermittent excursions than the other systems.

Many kinase inhibitors use hydrogen bonds to anchor ligands in the ATP-binding pocket and determine specificity. For GSK-3β, a strong hydrogen bond network helps stabilize the inhibitor’s conformation, reducing conformational entropy and increasing apparent affinity. Persistent hydrogen bonds can also enable crucial polar interactions that withstand solvent competition and keep the ligand catalytically relevant over inhibitory periods. Counting and characterizing protein-ligand hydrogen bonds through MD trajectories is not just descriptive, but also links microscopic contact patterns to thermodynamic and structural signatures (fold stability, binding-pose retention, and selectivity) that determine GSK-3β inhibitor efficacy^60–62^.

**Fig. 10:**
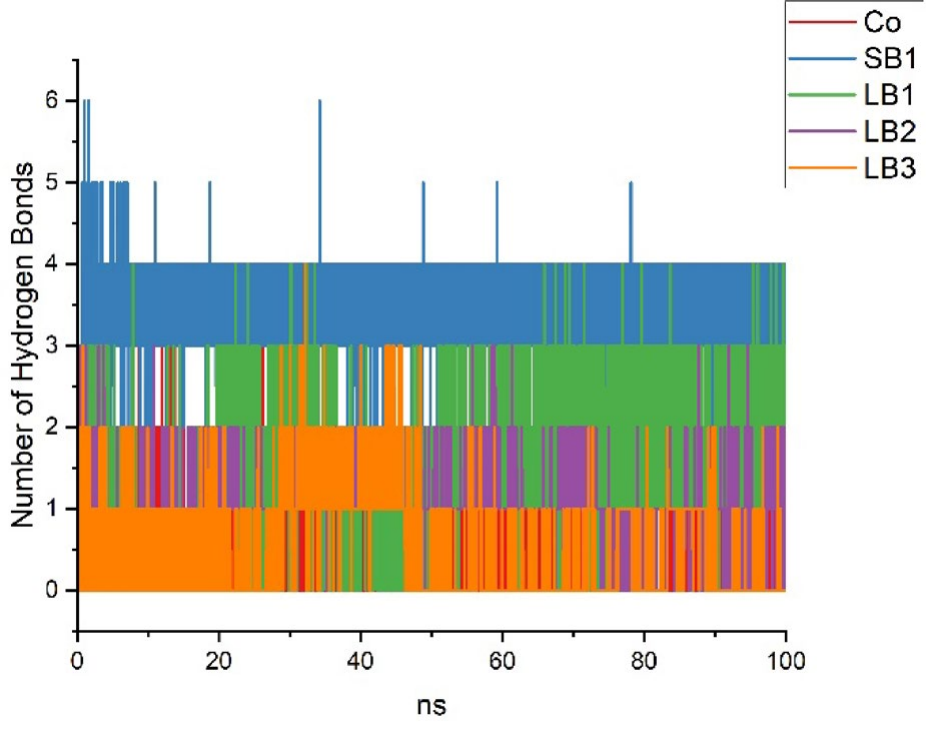
Number of hydrogen bonds generated by the two pharmacophore models and the co-crystallized ligand over the 100-ns production run.

H-bond time series indicate model-dependent changes that explain RMSD behavior (Fig. 10). The co-crystallized ligand has sparse hydrogen bonding (usually 0–2 H-bonds) while **SB1** generates a more persistent collection of polar contacts (3–6 H-bonds) during the run, indicating prolonged interactions. In contrast, **LB1** forms fewer H-bonds (usually 0–3). Still, these contacts appear more consistent during the production window, which matches its superior ligand RMSD (i.e., tighter pose retention) even when its backbone stabilization occurs later. This suggests **LB1** achieves pose stability through fewer well-maintained interactions than many transient ones. The H-bond counts fluctuate across all traces, with transient spikes and short-lived losses. **SB1**’s abundant H-bonding correlates with its early, low backbone RMSD, while **LB1**’s steadier (though fewer) H-bonds correlate with its lower ligand RMSD in the production period.

MD trajectories report radius of gyration (Rg) and solvent-accessible surface area (SASA) to quantify macromolecular compactness and solvent exposure. Small, low-variance Rg indicates that the system remains compact and that no large unfolding or domain-separation events occur during the simulation. In contrast, sustained increases or large fluctuations in Rg indicate the structure’s transient expansion or breathing motions. SASA measures the protein surface accessible to solvent and is sensitive to side-chain rearrangements, loop motions, and ligand repositioning. Increases in SASA indicate pocket opening or ligand egress, while decreases indicate burial or more intimate residue-ligand packing^63,64^.

**Fig. 11:**
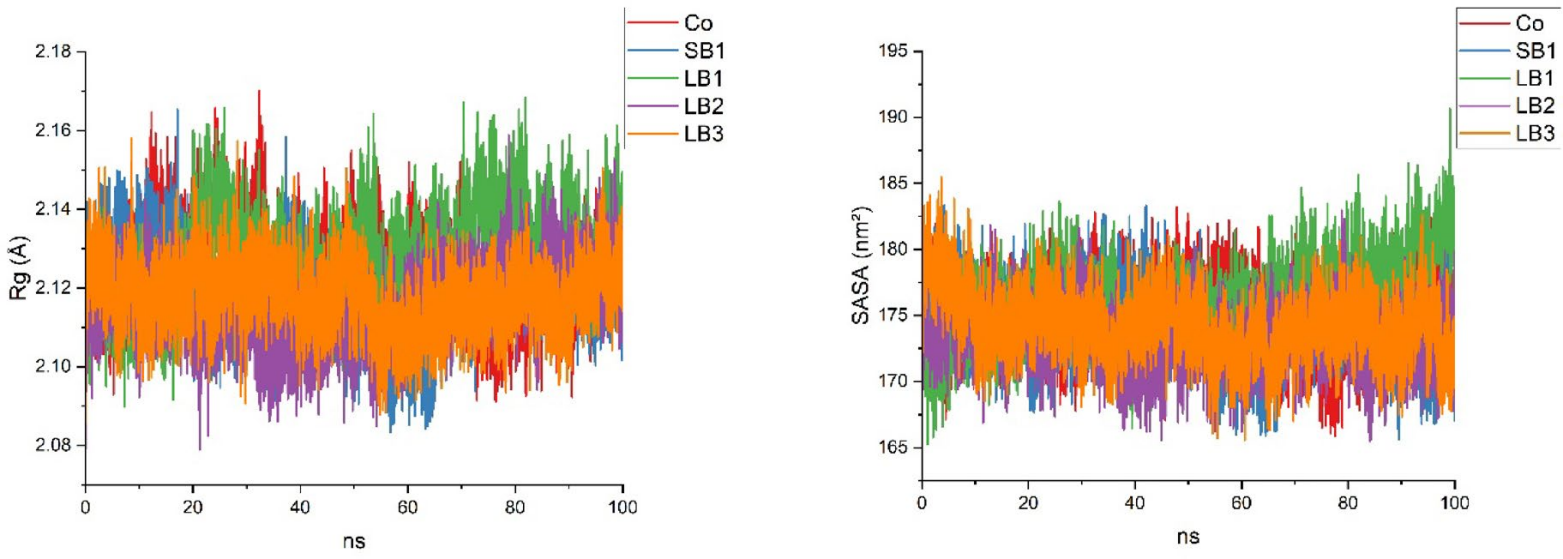
Rg **(left panel)** and SASA **(right panel)** values for the five trajectories.

Throughout all systems (**Co**, **SB1**, and **LB1-LB3**), the protein maintains a compact structure for most of the 100-ns trajectories (Fig. 11). The Rg values begin at approximately 2.08–2.09 Å and predominantly remain within a narrow range of 2.10–2.14 Å. The raw Rg traces exhibit only minor, rapid fluctuations on the order of 0.01–0.04 Å, suggesting the absence of global unfolding. Despite the overall stability, spikes are observed at several intervals, particularly around the ~ 28 ns region and in the ~ 54–58 ns window. These occurrences are consistent with short-lived breathing motions or local rearrangements, rather than a persistent loss of compactness. The SASA traces provide additional insights: initial SASA values exhibit minor variations across systems (e.g., **Co** ≈ 166.8 nm^2^ and **SB1** ≈ 170.1 nm^2^), while most trajectories fluctuate around ~ 170–179 nm^2^, with occasional peaks reaching ~ 181–184 nm^2^. This suggests intermittent increases in solvent exposure, such as pocket opening or side-chain reorientation, which temporally align with certain Rg excursions.

### Binding free energy calculations (MM/PBSA and MM/GBSA)

To provide a more rigorous thermodynamic validation of the binding affinities beyond the static docking scores, the Molecular Mechanics Poisson-Boltzmann Surface Area (MM/PBSA) and Generalized Born Surface Area (MM/GBSA) methods were employed. Binding free energies (ΔGbind) and their corresponding energy components were calculated over the stabilized production trajectories for the co-crystallized ligand **(Co)**, the ligand-based hit **(LB1)**, and the structure-based hit **(SB1)**. The energy decomposition analysis is summarized in Table 3. The calculations conclusively support the earlier docking and structural stability predictions. Both prioritized hits demonstrated notably stronger binding free energies compared to the co-crystallized reference. Under the MM/GBSA model, the co-crystallized ligand exhibited a ΔGbind of -8.75 ± 6.93 kcal/mol. In contrast, **LB1** and **SB1** displayed highly favorable binding energies of -25.74 ± 3.41 kcal/mol and − 27.68 ± 7.20 kcal/mol, respectively. A parallel trend was observed using the MM/PBSA method, confirming the robustness of these thermodynamic profiles.

Furthermore, the energy decomposition provides critical insights into the distinct binding mechanisms of the two candidates. The binding of **LB1** is predominantly driven by highly favorable van der Waals interactions (ΔEvdW = -36.79 ± 3.61 kcal/mol) and non-polar solvation energy (-4.74 ± 0.44 kcal/mol). Conversely, the exceptionally strong affinity of **SB1** is overwhelmingly dictated by massive electrostatic contributions (ΔEele = -214.36 ± 33.80 kcal/mol). This profound electrostatic stabilization of **SB1** perfectly corroborates our previous MD observations, specifically the formation of a robust and persistent network of 3–6 hydrogen bonds (Fig. 9) that tightly anchors the ligand within the ATP-binding cleft despite heavy polar solvation penalties. Together, these thermodynamic calculations validate the structural integrity observed during the MD simulations and strongly support the advancement of both **LB1** and **SB1** as potent GSK-3β inhibitors.

**Table 3:** Energy decomposition of MM/GBSA and MM/PBSA binding free energies. Values are in kcal/mol and reported as mean ± SD.

System	Method	ΔEvdW	ΔEele	Polar solvation	Non-polar solvation	ΔGgas	ΔGsolv	ΔGbind
Co	GB	-14.27 ± 9.22	-8.09 ± 8.55	15.57 ± 10.96	-1.96 ± 1.25	-22.36 ± 16.14	13.61 ± 9.88	-8.75 ± 6.93
Co	PB	-14.27 ± 9.22	-8.09 ± 8.55	16.61 ± 12.69	-1.82 ± 1.10	-22.36 ± 16.14	14.78 ± 11.72	-7.58 ± 5.32
LB1	GB	-36.79 ± 3.61	-12.18 ± 5.36	27.97 ± 4.89	-4.74 ± 0.44	-48.97 ± 6.85	23.23 ± 4.69	-25.74 ± 3.41
LB1	PB	-36.79 ± 3.61	-12.18 ± 5.36	30.56 ± 5.00	-3.83 ± 0.21	-48.97 ± 6.85	26.73 ± 4.92	-22.24 ± 3.91
SB1	GB	-9.83 ± 4.51	-214.36 ± 33.80	198.95 ± 26.66	-2.45 ± 0.66	-224.19 ± 32.84	196.50 ± 26.77	-27.68 ± 7.20
SB1	PB	-9.83 ± 4.51	-214.36 ± 33.80	199.65 ± 28.35	-2.31 ± 0.52	-224.19 ± 32.84	197.34 ± 28.48	-26.85 ± 5.43

### Validation of ligand-based and structure-based pharmacophore models

The validation of ligand-based and structure-based pharmacophore models was conducted using active-decoy datasets to assess their efficacy in distinguishing known GSK-3β active compounds from LUDe-generated decoys. ROC curve analysis utilized negative RMSD values as the ranking score, as lower pharmacophore RMSD values indicate superior alignment with pharmacophoric features. In the ligand-based pharmacophore model, the validation dataset included three active compounds and 150 decoy compounds. The model effectively identified 2 of the 3 active compounds and 7 of the 150 decoys. This resulted in a sensitivity of 66.67% and a specificity of 95.33%. ROC curve analysis yielded an AUC value of 0.8233, suggesting a strong preliminary discriminatory capability. The standard error was 0.1692, accompanied by a 95% confidence interval of 0.4917-1.000 and a p-value of 0.0555, which is considered borderline. These results indicate that the ligand-based pharmacophore model effectively identified active compounds while largely excluding decoys, demonstrating strong selectivity.

In the structure-based pharmacophore model, the validation dataset included one active compound and fifty decoys. The model identified the active compound along with 11 of the 50 decoys. This resulted in a sensitivity of 100% and a specificity of 78.00%. ROC curve analysis indicated an AUC value of 0.8200, suggesting satisfactory preliminary discriminatory performance. The standard error was 0.05433, with a 95% confidence interval ranging from 0.7135 to 0.9265, and a p-value of 0.2770. While the structure-based model effectively identified the active compound, the retrieval of 11 decoys suggests reduced selectivity in comparison to the ligand-based model.

**Fig. 12:**
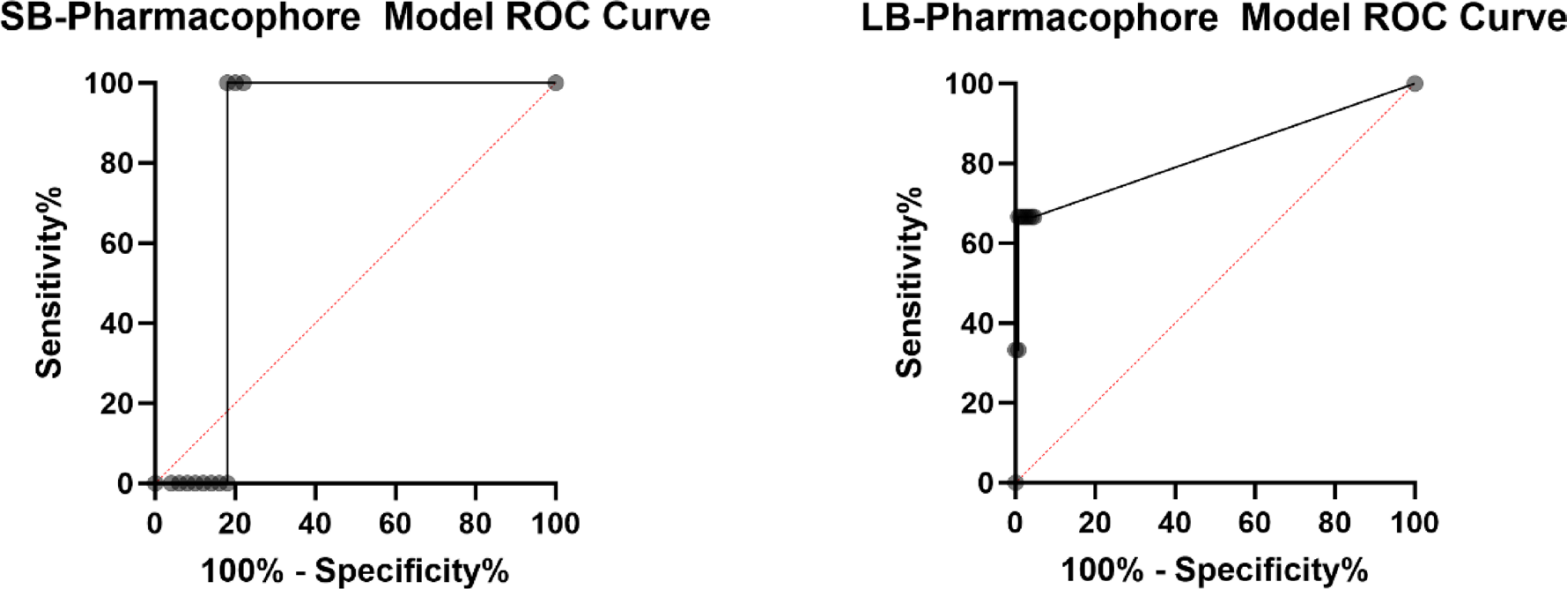
ROC curve analysis of the pharmacophore models.

Both pharmacophore models demonstrated AUC values exceeding 0.8, indicating their efficacy in differentiating active compounds from decoys. In contrast, the ligand-based model exhibited enhanced decoy rejection, retrieving merely 4.67% of the decoys, whereas the structure-based model retrieved 22.00%. Consequently, the ligand-based pharmacophore model demonstrated greater selectivity, whereas the structure-based pharmacophore model exhibited acceptable yet comparatively lower selectivity in preliminary performance (Fig. 12). Given the restricted availability of experimentally validated active compounds for validation, especially concerning the structure-based model, the ROC results must be regarded as preliminary retrospective validation. The acceptable AUC values obtained for both models validate their application in subsequent virtual screening, molecular docking, ADME/BBB filtering, toxicity prediction, and molecular dynamics simulation workflows.

## Discussion

This study integrates complementary ligand- and structure-based pharmacophore strategies with extensive database screening, docking, ADME filtering, toxicity prediction, and molecular dynamics simulations to identify and prioritize potential GSK-3β inhibitors for CNS applications. The two parallel pharmacophore approaches offered distinct yet complementary pathways into chemical space. The ligand-based pharmacophore model extracted conserved interaction geometries from seven known GSK-3β inhibitors, enabling a broad and chemotype-diverse search that identified 1,085 hits. In contrast, the structure-based pharmacophore model incorporated explicit protein-ligand interaction features derived from the GSK-3β crystal structure, resulting in a more focused set of 36 candidates. Applying a stringent pharmacophore RMSD cutoff of ≤ 1.0 Å effectively reduced the screened chemical space to manageable hit lists for subsequent docking and pharmacokinetic evaluation. These complementary strategies increased the likelihood of identifying both scaffold-diverse and protein-complementary binders, making the workflow suitable for early-stage discovery efforts aimed at CNS-relevant GSK-3β inhibitors.

To support the reliability of the generated pharmacophore hypotheses, retrospective ROC curve validation was incorporated using active-decoy datasets. For the ligand-based pharmacophore model, 3 experimentally reported active GSK-3β inhibitors and 150 LUDe-generated decoys were used. The model retrieved 2 out of 3 active compounds while retrieving only 7 out of 150 decoys, corresponding to 66.67% sensitivity and 95.33% specificity. ROC analysis produced an AUC value of 0.8233, indicating good preliminary discriminatory ability. Although the confidence interval was wide and the p value was borderline, this result is reasonable considering the limited number of available active compounds used for validation. Importantly, the low decoy retrieval rate indicates that the ligand-based pharmacophore model was selective and capable of rejecting most presumed inactive molecules.

The structure-based pharmacophore model was also retrospectively validated using 1 active compound and 50 LUDe-generated decoys. The model successfully retrieved the active compound together with 11 decoys, producing 100% sensitivity and 78.00% specificity. The corresponding ROC-AUC value was 0.8200, suggesting acceptable preliminary discriminatory performance. However, the higher decoy retrieval rate compared with the ligand-based model indicates that the structure-based pharmacophore was less selective. This may reflect the broader permissiveness of the protein-derived interaction pattern, where several decoys were still able to satisfy the essential spatial arrangement of the pharmacophoric features. Therefore, while both pharmacophore models achieved AUC values above 0.8, the ligand-based model showed superior decoy rejection and appeared more selective in the retrospective validation (Table 4).

**Table 4:** ROC-based validation results of the ligand-based and structure-based pharmacophore models.

Parameter	LB-pharmacophore model	SB-pharmacophore model
Number of active compounds	3	1
Number of decoys	150	50
Retrieved active compounds	2	1
Retrieved decoys	7	11
Sensitivity	66.67%	100%
Specificity	95.33%	78.00%
False-positive rate	4.67%	22.00%
AUC	0.8233	0.8200
Standard error	0.1692	0.05433
95% confidence interval	0.4917-1.0000	0.7135–0.9265
P value	0.0555	0.2770
Interpretation	Good preliminary discrimination with higher selectivity	Acceptable preliminary discrimination with lower selectivity

The ROC validation results strengthen the use of both pharmacophore models as initial virtual screening filters. Nevertheless, these findings should be interpreted cautiously because of the limited number of experimentally confirmed actives, particularly for the structure-based model. For this reason, pharmacophore validation was not considered as a standalone proof of biological activity, but rather as an early retrospective assessment that was integrated with additional downstream filters. The subsequent docking, ADME/BBB prediction, toxicity assessment, and molecular dynamics simulations collectively provided additional layers of validation and prioritization. This multi-stage strategy reduced the risk of relying exclusively on pharmacophore fit and helped identify candidates with more favorable binding, pharmacokinetic, and dynamic stability profiles.

After docking and triage, compounds exhibiting predicted binding energies superior to those of the co-crystallized reference ligand were prioritized. Of the ligand-based hits, 957 out of 1,085, and a notable number of structure-based candidates, 22 out of 36, exceeded the reference docking score of -6.80 kcal/mol. Among the compounds, the four leading candidates that met the ADME and BBB-related criteria, specifically **SB1** and **LB1**-**LB3**, were chosen for redocking, comprehensive interaction analysis, and molecular dynamics simulations. The redocking RMSD of 1.45 Å for the co-crystallized ligand validated the docking protocol’s reliability and affirmed the internal consistency of the docking poses utilized for comparison. The docking scores of the prioritized hits, which range from − 7.00 to -7.39 kcal/mol, indicate that the pharmacophore-guided workflow effectively enriched ligands with more advantageous docking profiles compared to the crystallographic ligand. These scores should be viewed as instruments for prioritizing hits rather than as definitive measures of absolute inhibitory potency, underscoring the necessity of subsequent dynamic analyses and future experimental validation.

A thorough analysis of the docking poses indicated interaction patterns that could elucidate the enhanced docking scores of the prioritized hits. The co-crystallized ligand predominantly established a hydrogen bond with Lys85, in addition to forming hydrophobic interactions. In contrast, several pharmacophore-derived hits demonstrated additional and more diverse anchoring interactions, including hydrogen bonds with key residues such as Lys183, Asn186, and Ser203, alongside π-anion interactions with Asp181 or Asp200 and π-cation interactions. Interactions in kinase binding sites are significant, as polar anchoring stabilizes ligand orientation within the ATP-binding cleft and minimizes unfavorable conformational freedom. Furthermore, electrostatic π-interactions may enhance enthalpic complementarity and contribute to the improved residence of the ligand within the binding pocket.

Molecular dynamics simulations offered a time-resolved validation layer that surpassed static docking methods. The 100-ns trajectories demonstrated that the majority of selected hits formed stable complexes with GSK-3β, as evidenced by acceptable backbone RMSD values and Rg/SASA profiles, which indicated no notable unfolding or ligand egress events. These observations affirm the structural integrity of the simulated complexes. **SB1** exhibited the lowest average backbone fluctuations and sustained a significant number of persistent hydrogen bonds, indicating early stabilization of the protein-ligand complex. **LB1** demonstrated consistently low ligand RMSD during the production period, suggesting effective pose retention despite a reduced number of hydrogen bonds formed. This finding indicates that a small number of strategically positioned interactions can maintain binding stability as effectively as a greater number of transient contacts.

The pharmacophore validation, docking, and molecular dynamics underscores the importance of an integrated computational workflow. Relying solely on docking energy may favor compounds whose perceived affinity is influenced by scoring artifacts or unrealistic static conformations. Pharmacophore screening alone may identify molecules that meet geometric criteria but may be inadequate regarding binding stability, blood-brain barrier permeability, or toxicity. By integrating ROC-validated pharmacophore screening with docking, ADME/BBB filtering, toxicity prediction, and molecular dynamics simulations, the current workflow systematically refined the initial hit lists and prioritized compounds exhibiting more balanced profiles^65,66^.

Table 5 summarizes the distinct computational profiles of **SB1** and **LB1**, which justify their selection as the most promising candidates. **SB1**, identified *via* the structure-based workflow, was characterized by its capacity to swiftly stabilize the kinase backbone, facilitated by a strong network of persistent hydrogen bonds. In contrast, the ligand-based hit **LB1** exhibited a marginally stronger predicted docking affinity and enhanced dynamic pose retention, maintaining a highly stable orientation with a ligand RMSD of approximately 0.721 Å, while depending on fewer but stable polar interactions. Together, the complementary strengths of **SB1**’s structural stabilization and **LB1**’s conformational persistence support the advancement of both compounds for future experimental validation as potential CNS-relevant GSK-3β inhibitors.

**Table 5:** Comparative summary of the top prioritized GSK-3β candidates.

Feature	Candidate SB1	Candidate LB1
Origin	Structure-based pharmacophore	Ligand-based pharmacophore
Predicted affinity	-7.16 kcal/mol	-7.28 kcal/mol
Dynamic pose retention	Moderate (Ligand RMSD ~ 1.115 Å)	Excellent (Ligand RMSD ~ 0.721 Å)
Backbone stability	Excellent (Early stabilization)	Good (Later stabilization)
Key interactions	3–6 persistent H-bonds	0–3 steady H-bonds

## Limitations

Retrospective ROC curve analysis was employed to assess the discriminatory capability of the pharmacophore models; however, validation was limited by the small number of experimentally confirmed active compounds. In the ligand-based model, validation was conducted using three active compounds and 150 decoys, whereas the structure-based model employed one active compound and 50 decoys. Consequently, although both models exhibited preliminary AUC values surpassing 0.8, the wide confidence intervals and non-significant p-values suggest limited statistical power. Moreover, the application of generated decoys assumes inactivity due to topological dissimilarity, an assumption that has yet to be experimentally validated. These factors, along with inherent limitations in pharmacophore screening platforms and their scoring procedures, require that these findings be viewed as preliminary retrospective validation rather than definitive evidence of model robustness.

Furthermore, the dependence on a singular 100-ns molecular dynamics trajectory for each system limits the statistical significance of the dynamic analyses. MD simulations exhibit significant sensitivity to initial conditions, and limited sampling durations may not sufficiently encompass the entire conformational phase space. Consequently, although these simulations serve as an essential tool for detecting unstable binding poses, conclusions about long-term complex stability remain predictive rather than thermodynamically definitive. Docking scores, MM/GBSA binding free-energy estimates, and in-silico ADMET parameters serve as tools for relative prioritization rather than as direct indicators of experimental potency. Ultimately, the predicted potency, binding modes, and pharmacokinetic profiles of candidates **SB1** and **LB1** necessitate thorough empirical validation. Enzymatic GSK-3β inhibition assays, cellular activity studies, and thorough in-vitro ADMET profiling are essential before progressing these scaffolds into medicinal chemistry optimization.

## Conclusions and future perspectives

In summary, the integrated protocol successfully enriched for GSK-3β binders and demonstrated that dynamic stability, rather than docking score alone, should inform hit prioritization. **SB1** and **LB1** are identified as the primary candidates for experimental validation: **SB1** is noted for its stability, confirmed by molecular dynamics, although solubility issues require further investigation. At the same time, **LB1** demonstrates a favorable combination of docking, molecular dynamics retention, and ADME properties. The subsequent steps should encompass biochemical inhibition assays, permeability and transporter evaluations, experimental solubility assessments, and focused medicinal chemistry to address any identified weaknesses, alongside enhanced free-energy calculations to improve potency rankings before initiating expensive experimental campaigns.

## Data Availability

Data generated or analyzed during this study are included in this published article. Other data will be made available on request.
